# M11‐CAR T cells inhibit murine cytomegalovirus *in vitro* upon recognition of virion‐ and cell‐associated glycoprotein B

**DOI:** 10.1002/cti2.70128

**Published:** 2026-11-03

**Authors:** Sabrina Kübel, Christopher Garçon, Hanna Menschikowski, Arne Cordsmeier, Jens‐Christian Albrecht, Friederike Oltmanns, Larissa Bauer, Manuel Hermann, Kristina Holdt, Heinrich Sticht, Marco Thomas, Thomas H Winkler, Brigitte Scholz, Doris Jungnickl, Armin Ensser

**Affiliations:** ^1^ Harald zur Hausen Institute of Virology University Hospital Erlangen, Friedrich‐Alexander‐Universität Erlangen‐Nürnberg Erlangen Germany; ^2^ Bioinformatics, Institute of Biochemistry Friedrich‐Alexander‐Universität Erlangen‐Nürnberg Erlangen Germany; ^3^ Chair of Genetics Friedrich‐Alexander‐Universität Erlangen‐Nürnberg Erlangen Germany; ^4^ Present address: C Garçon, Bavarian Nordic A/S Planegg Germany; ^5^ Present address: H Menschikowski, BG Trauma Centre Tübingen Eberhard Karls University Tübingen Tübingen Germany; ^6^ Present address: L Bauer, Max Planck Centre for Physics and Medicine Erlangen Germany

**Keywords:** chimeric antigen receptor, cytokines, cytomegalovirus, glycoprotein

## Abstract

**Objectives:**

Viral infections are predominantly managed with conventional antiviral drugs, which, despite their efficacy, can cause toxicity and promote resistance. Cell‐based immunotherapies offer an alternative, but cytomegaloviruses (CMV) profoundly interfere with MHC class I antigen presentation, thereby impairing conventional virus‐specific T‐cell responses towards infected cells. We therefore aimed to develop an MHC‐unrestricted CAR T‐cell approach for CMV infection and investigate its antiviral activity.

**Methods:**

We generated M11‐CAR, a fully murine, second‐generation CAR based on the M11 monoclonal antibody targeting glycoprotein B (gB) of murine CMV (MCMV). Primary splenocytes were transduced to assess CAR functionality following ectopic gB expression or MCMV infection. T‐cell cytotoxicity, cytokine production and antiviral activity were evaluated and compared with human CD19‐specific control CAR T cells.

**Results:**

M11‐CAR T cells specifically recognized gB following ectopic expression and MCMV infection, inducing cytotoxicity and cytokine production. CAR‐mediated cytokine production, particularly interferon‐γ, translated into robust MCMV inhibition. Increasing MCMV loads impaired cytokine responses of CD19‐CAR T cells, whereas M11‐CAR T cells maintained strong responses. This effect was partially attributable to direct recognition of virion‐associated gB, resulting in activation of M11‐CARs.

**Conclusions:**

M11‐CAR T cells enable MHC‐independent recognition of MCMV, providing a potential strategy to circumvent CMV‐mediated disruption of antigen presentation and immune recognition. Our findings demonstrate that virions can directly shape virus‐specific CAR T‐cell responses and highlight the importance of antigen recognition mechanisms that are independent of MHC presentation. This provides a basis for refining CAR designs for persistent viral infections characterized by immune evasion.

## Introduction

Our immune system continuously discriminates between self and foreign to sense pathogens and to protect us from bacterial and viral infections.^1^ Viruses such as the Human Immunodeficiency Virus 1 (HIV‐1), the Epstein–Barr Virus (EBV) and the Human Cytomegalovirus (HCMV) have developed a variety of mechanisms to escape immune cell recognition and subsequent elimination.^2^ For instance, among its various immune evasion strategies, HCMV masters the escape from T cells and natural killer (NK) cells by interfering with the expression of major histocompatibility complexes (MHC) and by encoding an NK ligand mimicry as well as a human interleukin (IL)‐10 homologue.^3^, ^4^, ^5^, ^6^ Combined, these immune evasion mechanisms mitigate the HCMV‐specific immune response and are key to the establishment of persistent infection.

Since Balfour nicknamed HCMV ‘troll of transplantation’ five decades ago,^7^ it remains a major contributor to morbidity and mortality among transplant recipients.^8^ Early after haematopoietic stem cell transplantation (HCT), immune control of latent HCMV becomes leaky as donor cells are not yet efficiently engrafted, posing the risk of HCMV DNAemia. Despite sophisticated pre‐emptive, prophylactic and therapeutic strategies, conventional antiviral drugs often fail to control DNAemia, culminating in HCMV disease. For instance, implementation of the viral terminase inhibitor Letermovir as a prophylactic drug has drastically reduced clinically significant HCMV disease in patients early after HCT^9^; however, it has not translated into improved overall survival of HCT patients.^10^ This has, among others, been attributed to an impaired reconstitution of CMV‐specific T cells under Letermovir, increasing the risk of HCMV reactivation after treatment termination.^11^, ^12^

In recent years, virus‐specific T (VST) cells have emerged as a promising strategy to restore cellular immunity: Upon ex vivo expansion and early after HCT, VST cells are adoptively transferred.^13^ VST cells facilitate antigen‐specific immunity via binding of their T‐cell receptor (TCR) to peptide‐loaded MHC. Clinical trials have been conducted and while study criteria vary, promising response rates and low adverse effects have been reported.^14^ Nevertheless, targeting HCMV with immunotherapies reliant on MHC presentation remains susceptible to viral interference with host cell MHC expression.^15^ A strategy circumventing MHC‐restricted T‐cell activation are chimeric antigen receptors (CAR). Relying on the binding properties of an antibody, these receptors redirect T cells to achieve an antigen‐specific and MHC‐independent T‐cell response. CAR T cells have been successfully introduced as immunotherapies for cancer and autoimmune disease.^16^, ^17^ In the context of chronic EBV or HIV‐1 infection, phase I and II clinical studies using virus‐specific CAR T cells are ongoing (e.g. NCT05587543, NCT06252402).^18^ In 2010, our research group established the first HCMV‐specific CAR targeting glycoprotein B (gB).^19^ Later, Olbrich et al. indicated a therapeutic potential of high‐affinity HCMV gB‐specific CAR T cells in a humanized mouse model.^20^ Humanized mouse models enable *in vivo* studies of human‐specific viruses and are important tools in immunovirology, yet their scientific significance can be limited due to species‐specific differences and inadequate modelling of systemic disease. To this day, no in‐human studies have been initiated for HCMV‐specific CAR T cells, underscoring the need for further preclinical refinement based on *in vitro* and *in vivo* experimental systems.

Bootz and colleagues investigated the therapeutic potential of monoclonal antibodies (mAb) targeting murine cytomegalovirus (MCMV) glycoproteins in an immunosuppressed MCMV mouse model.^21^ They showed that *in vivo* treatment with the gB‐specific mAb M11 significantly reduces organ viral load. These results provide the rationale for engineering an MCMV gB‐targeting CAR that links M11‐based antigen recognition to T‐cell effector functions. Here, we describe the construction of a fully murine second‐generation M11‐based CAR construct incorporating the murine 4‐1BB costimulatory domain and characterize its specificity for MCMV gB as well as its antiviral activity *in vitro*.

## Results

### Monoclonal antibody M11 facilitates recognition of cell‐associated glycoprotein B (m55)

MCMV157luc reporter virus is a well‐established system that facilitates quantification and imaging, providing a reliable direct correlate of viral replication and spread.^21^, ^22^, ^23^, ^24^, ^25^ Bootz et al. infected C57BL/6 mice with the MCMV157luc reporter virus, boosted the immune response with irradiated virions and obtained MCMV‐specific murine B cells from spleens 4 weeks after infection.^21^ Hybridoma cells were generated and characterized for antigen specificity. The mAb M11 was identified as an MCMV gB‐specific antibody that binds to the antigen region 4 (AD‐4). AD‐4 is predicted to be accessible in both the pre‐ and postfusion conformation of trimeric gB, making M11 an attractive candidate for MCMV‐specific CAR design (Figure 1a and b). Therefore, we analysed gB expression on RNA and protein level and characterized M11‐mediated gB recognition throughout the MCMV replication cycle.

**Figure 1:**
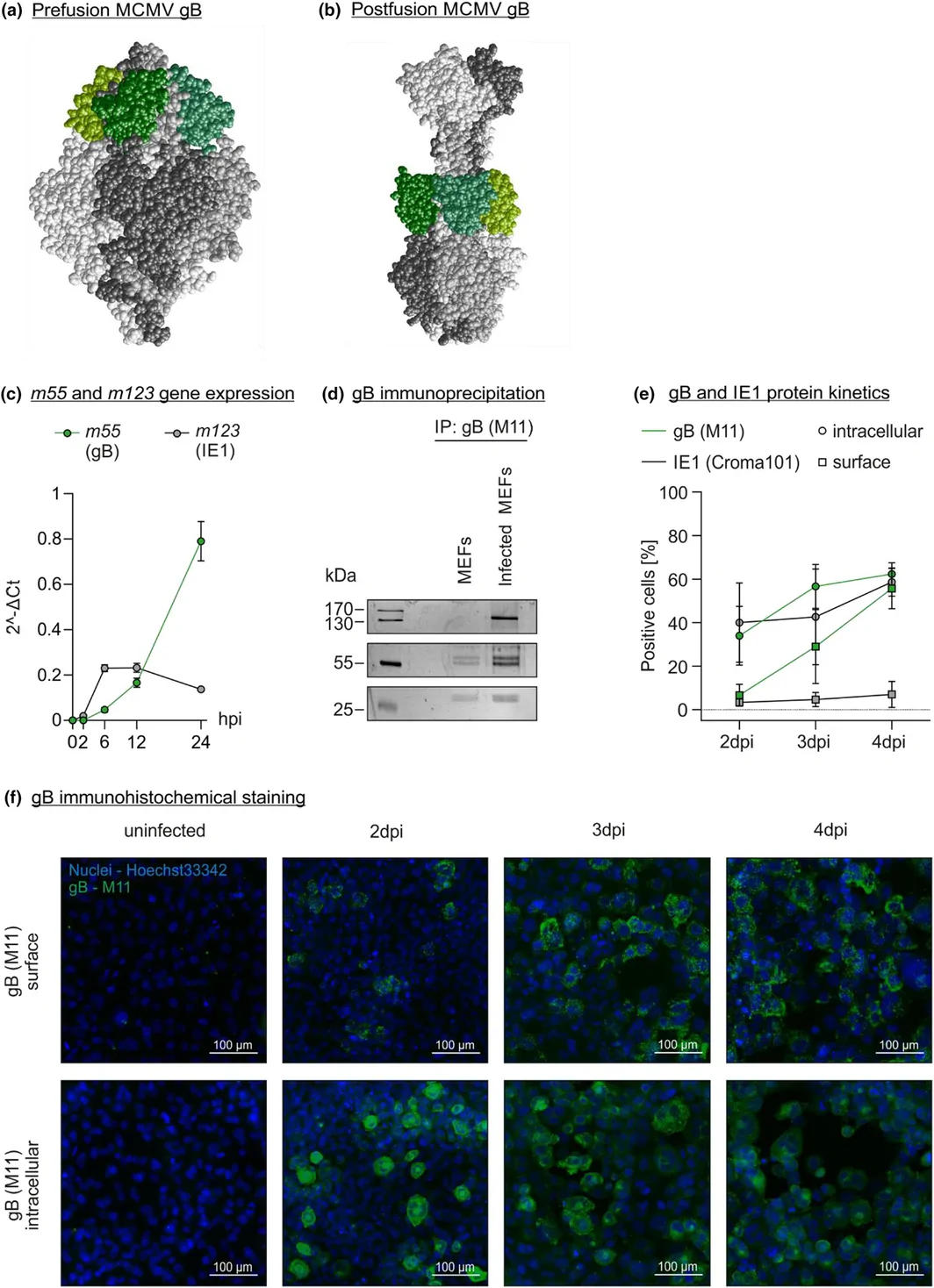
MCMV gB and its recognition by mAb M11. **(a, b)** 3D model of the trimeric prefusion and postfusion conformation of MCMV gB, based on HCMV gB (PDB: 7KDP^71^ and 5XCF^72^). Protomers are shown in black, light and dark grey. M11 targets the antigen‐domain 4 (AD‐4), which corresponds to the structural domain II and is highlighted in green. **(c)** Relative *m55* (gB) and *m123* (IE1) expression (2^−∆Ct^, normalized to *Hrpt1*, *n* = 3, mean ± SD) in MCMV157luc‐infected murine embryonic fibroblasts (MEFs). hpi, hours post infection. **(d)** Coomassie‐stained gel of proteins recovered in the M11‐immunoprecipitation (IP) using lysates of uninfected or MCMV157luc‐infected MEFs. kDa, kilodalton. **(e)** Mean percentage ± standard deviation of surface and intracellular gB (M11)‐ or IE1 (Croma101)‐positive MEFs during the course of an MCMV157luc infection, analysed by immunohistochemical staining (*n* = 3). dpi, days post infection. **(f)** Representative images of gB (M11) surface and intracellular staining of MCMV157luc‐infected MEFs.

First, we performed real‐time quantitative polymerase chain reaction (RT‐qPCR) experiments to investigate *m55* transcription which encodes for MCMV gB. Within 24 h, we detected a sharp increase in *m55* transcripts in MCMV157luc‐infected murine embryonic fibroblasts (MEFs) (Figure 1c). We additionally analysed immediate early protein (IE1; *m123*) gene expression as a control for successful MCMV infection. Next, we performed immunoprecipitation assays (IPs) to confirm gB expression and M11 binding on protein level. Therefore, we incubated M11‐containing hybridoma supernatant with lysates of infected or uninfected MEFs (4 days post infection (dpi), multiplicity of infection (MOI) 4). M11‐gB antibody‐protein complexes were purified using pan mouse immunoglobulin G (IgG) beads, separated by electrophoresis and analysed by Coomassie brilliant blue staining (Figure 1d, Supplementary figure 1). Both IPs displayed bands corresponding to heavy (~55 kilo Dalton (kDa)) and light chains (~25 kDa) of M11; however, only the IP with lysate of infected MEFs resulted in a strong 130 kDa signal, consistent with the specific signal for MCMV gB reported by Rapp et al. in 1992.^26^

Finally, we performed immunohistochemical staining of MCMV157luc‐infected MEFs with M11‐ or Croma101‐containing hybridoma supernatant to characterize total and surface expression of gB and IE1, respectively (Figure 1e and f, Supplementary figure 2). Staining of total protein expression was achieved using a saponin‐containing buffer, hereinafter referred to as ‘intracellular’ staining. mAb Croma101 specifically binds IE1 which is exclusively localized in the nucleus of infected cells. Over the course of infection, we detected an increase in the percentage of Croma101‐positive and thus MCMV‐infected MEFs (~40% to ~59%) by intracellular staining, while cells remained negative in the Croma101‐surface staining. In contrast, we observed a strong increase in M11 and thus gB‐positive cells in both the intracellular (~34% to ~62%) and surface staining (~7% to ~56%).

Our data demonstrate abundant transcription and translation of *m55* during an MCMV infection as well as binding of the mAb M11 to cell‐associated gB, suggesting that an M11‐based CAR can redirect T‐cell effector functions to MCMV‐infected and gB‐displaying cells.

### Generation and functional characterization of M11‐ and FMC63‐based CAR T cells

Retroviral expression vectors were produced to generate second‐generation M11‐ and FMC63‐based CAR T cells (Figure 2a). In this study, the anti‐human CD19 (hCD19)‐specific FMC63‐CAR functioned as a virus‐independent control. Single chain variable fragments (scFv) were designed in silico in V_L_ > V_H_ orientation with a glycine‐serine linker ((G_4_S)_3_) based on the sequences of heavy and light chain variable regions (V_H_, V_L_) of mAb M11 or FMC63.^27^ Both scFvs were synthesized de novo and cloned into a vector containing a fully murine second‐generation CAR backbone: a CD8α hinge and transmembrane domain (TMD) and the intracellular domain (ICD) of 4‐1BB and CD3ζ. A Flag tag was incorporated between scFv and hinge domain to facilitate CAR surface detection using an anti‐Flag antibody (Ab). The final CAR construct was cloned into the pMSCV vector, allowing retroviral transduction and subsequent puromycin (puro) selection of transduced cells.^28^

**Figure 2:**
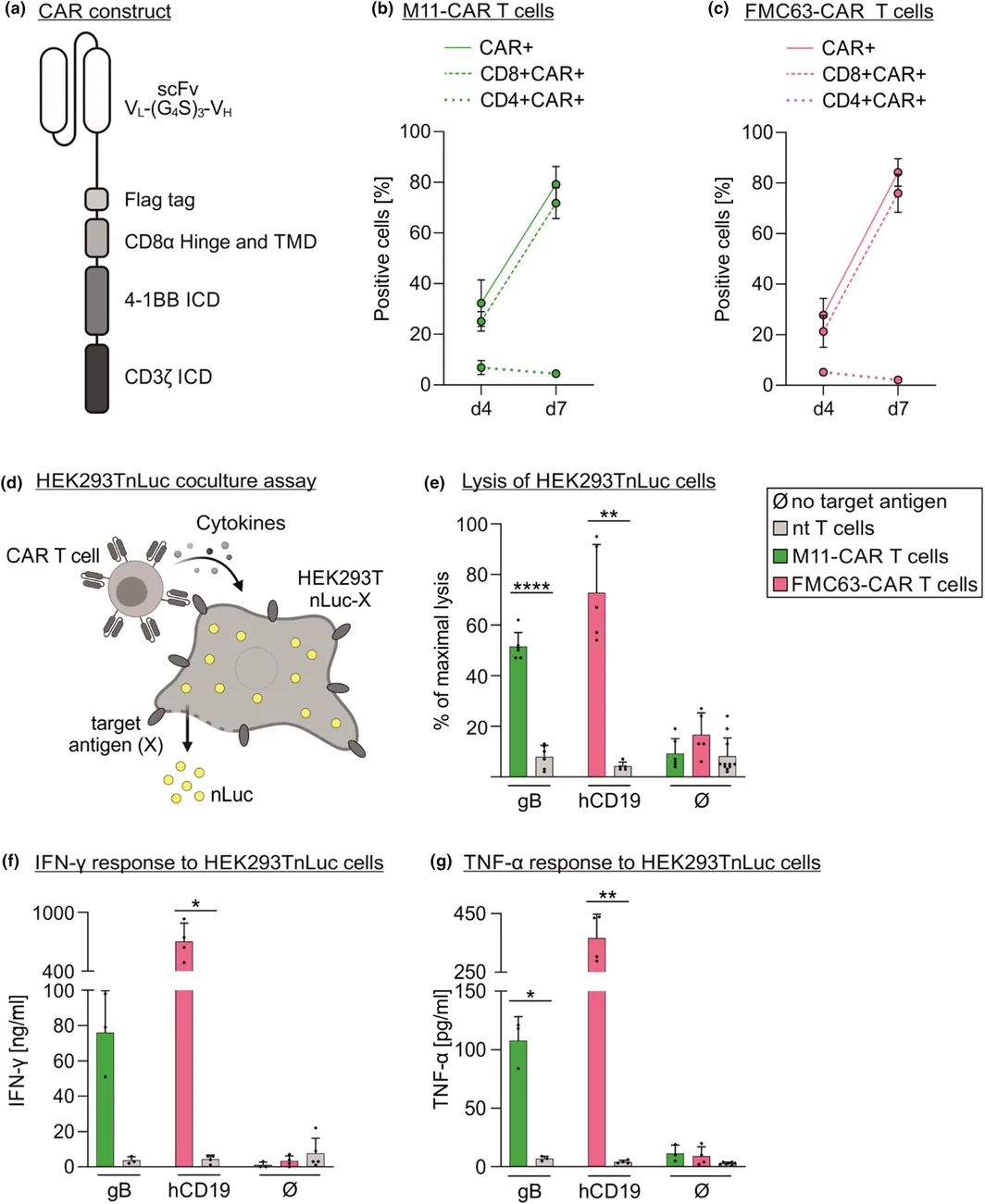
M11‐ and FMC63‐based CAR constructs mediate target antigen‐specific T‐cell effector functions. **(a)** Scheme of our CAR constructs. Created with BioRender. (G_4_S)_3_, (Glycin_4_Serine)_3_‐Linker; ICD, intracellular domain; scFv, single chain variable fragment; TMB, transmembrane domain; V_H_, heavy chain variable region; V_L_, light chain variable region. **(b, c)** Flow cytometric analysis of CAR, as well as CD8 and CD4 surface expression, on M11 or FMC63 CAR T cells (*n* = 4, mean ± SD). d, day. **(d)** Scheme of the HEK293TnLuc coculture assay. HEK293TnLuc cells with or without target antigen expression are cocultured with CAR T cells for 24 h. CAR‐mediated target recognition induces T‐cell effector functions. Release of Nano‐Luciferase (nLuc) into the coculture supernatant correlates with cell lysis. Created with BioRender. **(e)** CAR‐mediated lysis of HEK293TnLuc cells. HEK293TnLuc‐gB (gB), HEK293TnLuc‐hCD19 (hCD19) or HEK293TnLuc (Ø) were cocultured with M11‐CAR (green, *n* = 5), FMC63‐CAR (pink, *n* = 5) or non‐transduced (nt; grey, *n* = 5–11) T cells. nLuc activity in the coculture supernatant was normalized to saponin‐induced maximum lysis of the respective HEK293TnLuc cell line. **(f, g)** CAR‐mediated IFN‐γ and TNF‐α production in HEK293TnLuc assay. Respective HEK293TnLuc cells were cocultured with M11‐CAR (green, *n* = 3), FMC63‐CAR (pink, *n* = 4) or nt (grey, *n* = 3–7) T cells. Supernatant cytokine levels were evaluated by ELISA. **(e–g)** Bars reflect biological replicates as individual data points and show mean ± standard deviation. A paired *t*‐test, followed by Holm‐Šídák correction was performed to compare CAR T‐cell treatment with the respective nt T‐cell group (*P ≤ 0.05, **P < 0.01, ****P < 0.0001).

In order to generate murine CAR T cells, we established a retroviral transduction protocol for murine splenocytes (Supplementary figure 3).^28^ Briefly, murine splenocytes are isolated and activated for 24 h with anti‐CD3‐ and anti‐CD28‐antibodies. CAR‐encoding ecotropic retroviruses are produced in Platinum‐E cells and used for transduction of activated splenocytes. CAR surface expression is determined on day 4 and 7 using flow cytometry (Figure 2b and c, Supplementary figure 4). Within 3 days of puro selection and T‐cell expansion (IL‐7, IL‐15), our protocol yields a high amount of murine CAR+ CD8+ T cells (~72% M11‐CAR, ~76% FMC63‐CAR). This protocol enables the generation of murine CAR T cells and thereby facilitates the functional assessment of the M11‐based CAR construct. Of note, non‐transduced (nt) murine T cells were required as controls in the following functional assays. Therefore, a proportion of each donor's splenocytes was processed in parallel but was not subjected to retroviruses and selection. Next, we assessed the functionality of the generated M11‐ and FMC63‐based murine CAR T cells in coculture assays with target‐expressing HEK293TnLuc reporter cells (Figure 2d).^28^, ^29^ We added murine CAR T cells or nt T cells 24 h after target cells had been seeded. On the next day, Nano‐Luciferase (nLuc) activity was measured in the coculture supernatant as a correlate for target cell lysis. More precisely, we evaluated lysis efficacy in comparison to saponin treatment, representing complete lysis. The M11‐ and FMC63‐CAR mediated efficient lysis of gB‐ or hCD19‐HEK293TnLuc cells, with a mean lysis of ~52% and ~73%, respectively (Figure 2e). To prove that the lysis is target‐specific and CAR‐mediated, CAR T cells were cocultured with reporter cells that do not express a target antigen. No significant lysis was detected in this control setting. As an additional correlate for T‐cell activation, IFN‐γ and tumor‐necrosis‐factor‐α (TNF‐α) concentrations were measured in the coculture supernatant via enzyme‐linked immunosorbent assay (ELISA; Figure 2f and g). Both CAR constructs mediate a significant and robust production of IFN‐γ and TNF‐α in the presence of their respective target. In control settings, only baseline cytokine concentrations were detected.

Together, these data demonstrate that target‐specific binding of the M11‐ and FMC63‐based CAR initiates signal transduction and subsequent T‐cell effector functions (e.g. cytotoxicity, cytokine secretion).

### CAR‐mediated inhibition of MCMV157luc *in vitro*

In order to investigate M11‐ and FMC63‐CAR T cells in the context of an MCMV infection, we utilized hCD19‐MEFs and the MCMV157luc reporter virus. In this experimental setting, FMC63‐CAR T cells are activated by ubiquitously expressed hCD19 (Figure 3a) while M11‐CAR T‐cell activation is restricted to gB‐surface positive MCMV157luc‐infected cells (Figure 3b). As indicated by our immunohistochemical staining, MCMV gB‐surface expression depends on the phase of infection (Figure 1f). Cells infected with the MCMV157luc reporter virus co‐express the firefly luciferase (Fluc). Accordingly, high luminescence signals can be detected in lysates of MCMV157luc‐infected hCD19‐MEFs (Figure 3c). Here, Fluc signal strength directly correlates with the initial MOI and thus viral load.^23^ This correlation is also detectable in the supernatant of infected hCD19‐MEFs, although the Fluc signal is ~3‐log less intense, indicating that only a small proportion is released into the supernatant, despite an active MCMV infection and high intracellular Fluc levels (Figure 3d). Acknowledging these findings, the MCMV157luc reporter virus allows two distinct readouts: (I) assessing total viral load, by analysing Fluc in the cell lysate and (II) determining lysis of infected cells, by measuring Fluc in the supernatant. We therefore infected hCD19‐MEFs with MCMV157luc (MOI 0.5–8) for 2 days, followed by 24 h coculture with nt, M11‐CAR or FMC63‐CAR T cells, and analysed Fluc activity in the cell lysate and supernatant. Under these conditions, it became evident that coculture with both CAR T cells, but not with nt T cells, results in a strong reduction of lysate Fluc levels and thus of MCMV157luc load (Figure 3e). Considering Fluc supernatant levels, we detected elevated levels in the coculture of infected MEFs with M11‐CAR, but not with FMC63‐CAR or nt T cells (Figure 3f). Notably, M11‐ and FMC63‐CAR T cells remained effective across all tested conditions; however, their viral inhibition and lysis efficacy declined with increasing initial MCMV157luc MOI (Figure 3e and f).

**Figure 3:**
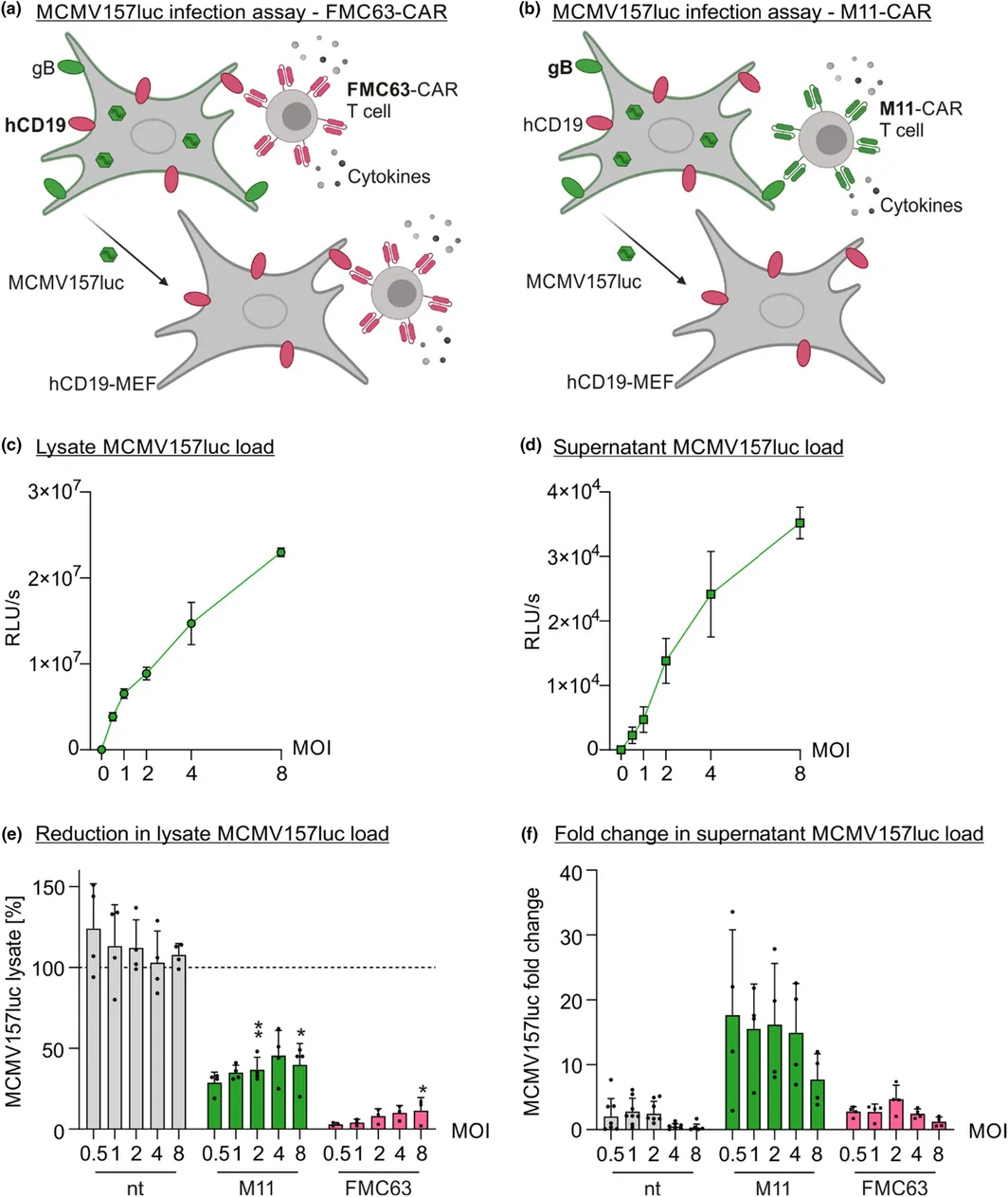
M11‐based CAR T cells inhibit MCMV157luc *in vitro*. **(a, b)** Simplified scheme of the MCMV157luc coculture assay. MCMV157luc‐infected murine embryonic fibroblasts (MEFs) expressing human CD19 (hCD19) are cocultured with non‐transduced (nt), FMC63‐CAR (A) or M11‐CAR T cells (B). CAR‐mediated target recognition induces T‐cell effector functions. Glycoprotein B (gB) surface expression is restricted to MCMV157luc‐infected cells. MCMV157luc‐infected MEFs express firefly luciferase (Fluc). Created with BioRender. **(c, d)** Relative light units per second (RLU/s), reflecting MCMV157luc load in lysate **(c)** or supernatant **(d)** of two‐day infected hCD19‐MEFs (RLU/s; *n* = 3, mean ± SD) in the absence of CAR‐T cells. **(e)** Changes in lysate MCMV157luc load upon coculture of infected hCD19‐MEFs with nt (*n* = 4), M11‐ (*n* = 4) or FMC63‐CAR T cells (*n* = 3). Fluc activity was measured 24 h after coculture. The obtained RLU/s values were normalized to the respective medium control (dashed line). **(f)** Fold change of supernatant MCMV157luc load upon coculture of infected hCD19‐MEFs with nt (*n* = 8), M11‐ (*n* = 4) or FMC63‐CAR T cells (*n* = 4). Fluc activity was measured 6 h after coculture. Obtained RLU/s values were used to calculate the fold change to the respective medium control, which was set to 1. **(e, f)** Bars reflect biological replicates as individual data points and show mean ± standard deviation. Paired *t*‐test, followed by Holm‐Šídák correction, was performed to compare CAR T‐cell treatment with respective nt T‐cell group (*P ≤ 0.05, **P < 0.01).

These findings demonstrate that M11‐based CAR T cells potently inhibit MCMV157luc *in vitro* in response to physiological MCMV gB expression and indicate CAR‐mediated lysis of infected cells.

### CAR‐dependent MCMV157luc inhibition is mediated by soluble factors such as IFN‐γ

Besides cytotoxicity, CAR T‐cell functionality is also defined by cytokine secretion. Thus, we investigated IFN‐γ and TNF‐α production in the MCMV157luc infection assay. Briefly, hCD19‐MEFs were infected with MCMV157luc for 2 or 5 days prior to 24 h coculture with nt, M11‐CAR or FMC63‐CAR T cells. Supernatant cytokine levels were determined by ELISA. Only baseline cytokine production was detected in no T cell and nt T‐cell conditions, whereas M11‐CAR and FMC63‐CAR T cells elicited a pronounced IFN‐γ and TNF‐α response (Figure 4a and b, Supplementary figure 5). The highest cytokine levels were detected in FMC63‐CAR T‐cell coculture settings, reflecting CAR‐mediated T‐cell activation by recognition of the ubiquitous and abundant hCD19 target antigen. Interestingly, a significant reduction of FMC63‐CAR IFN‐γ response was observed with increasing MOI (Figure 4a). The opposite trend was detected for M11‐CAR T cells: supernatant IFN‐γ and TNF‐α levels increased with duration of MCMV157luc infection and MOI. In particular, the TNF‐α response of M11‐CAR T cells showed a clear MOI dependency (Figure 4b, Supplementary figure 5). Although cytokine levels were lower than those induced by the hCD19‐specific FMC63 control CAR, these data demonstrate that M11‐CAR T cells mount a robust cytokine response to MCMV gB expressed during MCMV infection.

**Figure 4:**
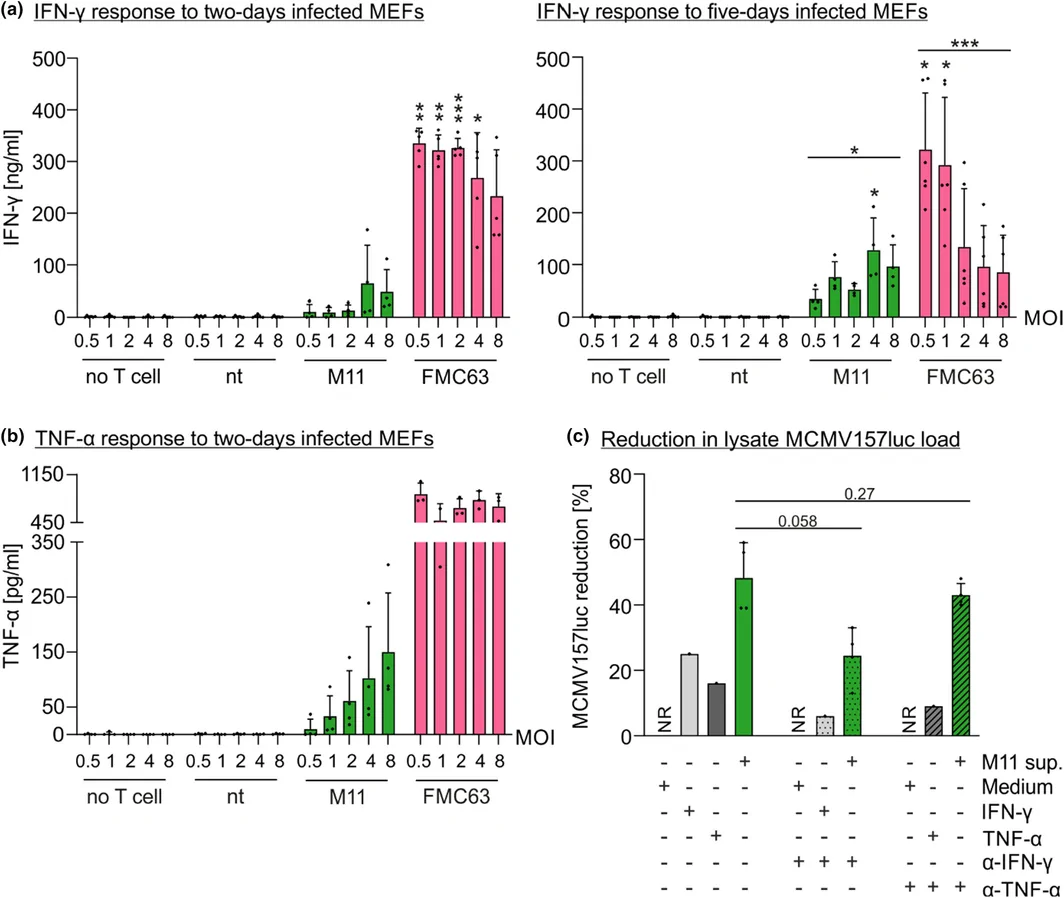
CAR‐mediated MCMV157luc inhibition is IFN‐γ dependent. **(a, b)** Supernatant IFN‐γ and TNF‐α concentrations in coculture of MCMV157luc‐infected hCD19‐MEFs with nt (*n* = 4–6), M11‐ (*n* = 4) or FMC63‐CAR T cells (*n* = 2–6). No T‐cell treatment served as a control. hCD19‐MEFs were infected for 2 or 5 days prior to coculture with increasing MOI (0.5–8). Bars reflect biological replicates as individual data points and show mean ± standard deviation. A paired *t*‐test, followed by Holm‐Šídák correction, was performed to compare CAR T‐cell treatment with respective nt T cell (asterisk above bar) as well as response to MOI 0.5 and MOI 8 within one group (asterisk above line; *P ≤ 0.05, **P < 0.01, ***P < 0.001). **(c)** Reduction in lysate MCVM157luc load mediated by M11‐CAR T‐cell supernatant. M11‐CAR T cells (*n* = 4, mean ± SD) were cocultured with MCMV157luc‐infected MEFs for 24 h. Cell‐free M11‐coculture supernatant (M11‐sup.) or medium was transferred to infected MEFs (2 days, multiplicity of infection (MOI) 0.5). Recombinant IFN‐γ, TNF‐α or neutralizing IFN‐γ and TNF‐α antibodies (α‐IFN‐γ, α‐TNF‐α) were supplemented as indicated. After 48 h incubation, lysate Fluc activity was measured. The obtained RLU/s values were normalized to the respective medium control, indicated by ‘no reduction’ (NR). Values above lines reflect paired *t*‐test, followed by Holm‐Šídák correction between treatment with M11‐sup. and M11‐sup. supplemented with a neutralizing mAb.

To link the M11‐CAR T‐cell cytokine response to the observed *in vitro* MCMV157luc inhibition, we performed a supernatant transfer assay. Here, M11‐CAR T cells were cocultured with MCMV157luc‐infected MEFs (two dpi, MOI 4) for 24 h, and the cell‐free, cytokine‐containing supernatant (M11 sup.) was collected. Subsequently, a new plate of infected MEFs (two dpi, MOI 0.5) was treated with either M11 sup., recombinant IFN‐γ or TNF‐α. Fluc activity was measured in the cell lysate after 48 h, and the reduction of viral load was calculated in comparison to the respective medium control. Transfer of the cell‐free M11 sup. was sufficient to reduce MCMV157luc viral load by ~48% (Figure 4c; green bar) and thus elicited a greater antiviral effect than the addition of 30 ng recombinant IFN‐γ (light grey bar; ~25% reduction) or 10 ng recombinant TNF‐α (dark grey bar; ~16% reduction) per well. To further assess the contribution of these cytokines to the pronounced M11 sup.‐mediated antiviral effect, IFN‐γ‐ or TNF‐α‐neutralizing Ab were added in parallel experiments (Figure 4c; α‐IFN‐γ, middle panel dotted bars; α‐TNF‐α, right panel dashed bars). Addition of α‐IFN‐γ or α‐TNF‐α to MEFs treated with the respective recombinant cytokine diminished the observed antiviral effect, highlighting the contribution of both cytokines to MCMV inhibition *in vitro*. In the presence of M11 sup., only addition of neutralizing IFN‐γ Ab, but not TNF‐α Ab, reversed the antiviral effect and partially restored viral spread. These results suggest that soluble factors—particularly IFN‐γ—contribute substantially to the MCMV inhibition facilitated by the M11‐CAR.

### M11‐CAR mediated recognition of virion‐associated gB contributes to cytokine response

M11‐CAR and FMC63‐CAR T cells show different cytokine responses to MCMV157luc‐infected MEFs. Given M11's capacity to neutralize MCMV,^21^, ^30^ we examined whether binding to virion‐associated gB shapes the M11‐CAR T cell response. First, we analysed the viral load in the supernatant of MCMV157luc‐infected hCD19‐MEFs by qPCR and detected an increasing level of viral DNA with increasing MOI (Figure 5a). Therefore, we hypothesized that M11‐CAR T cells encounter increasing levels of viral particles in our MCMV157luc infection assay. Thus, we incubated nt, M11‐CAR or FMC63‐CAR T cells with increasing MOI of cell‐free, sucrose‐gradient purified MCMV157luc virions for 24 h (Figure 5b). α‐CD3‐ and α‐CD28‐Ab served as stimulation control (ctrl), and T‐cell cytokine response was measured. The M11‐based CAR mediates a robust T‐cell response upon binding to cell‐free MCMV157luc virions, as evident from MOI‐dependent IFN‐γ and TNF‐α production (Figure 5c and d). Only baseline cytokine levels were detected for nt or FMC63‐CAR T cells upon incubation with MCMV157luc virions, while all cells responded to the ctrl stimuli.

**Figure 5:**
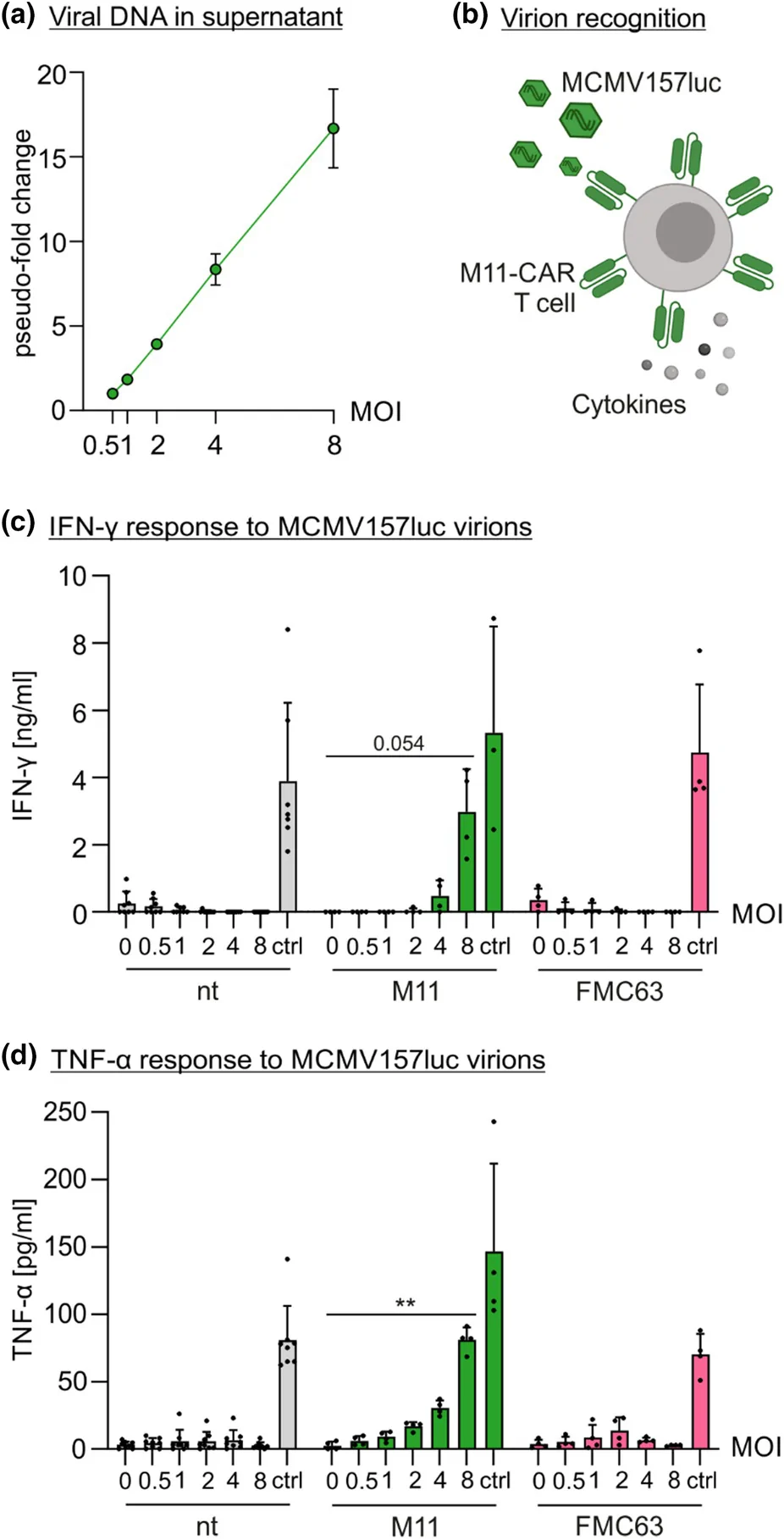
Recognition of MCMV virion‐associated gB by M11‐based CAR T cells. **(a)** Semi‐quantitative qPCR of MCMV157luc viral DNA in the supernatant of 2‐day MCMV157luc‐infected MEFs. Pseudo‐fold change of *m123* cycle threshold (Ct) relative to infection with multiplicity of infection (MOI) 0.5 (*n* = 4, mean ± SD). **(b)** Scheme of MCMV157luc virion recognition by M11‐CAR T cells. Created with BioRender. **(c, d)** IFN‐γ and TNF‐α response of nt (*n* = 6–8), M11‐CAR (*n* = 3–4) or FMC63‐CAR (*n* = 4) T cells to MCMV157luc virions (MOI 0.5–8) or anti‐CD3ε and anti‐CD28 antibodies (ctrl). Cytokine levels were analysed after 24 h incubation by enzyme‐linked immunosorbent assay. Bars reflect biological replicates as individual data points and show mean ± standard deviation. A paired *t*‐test, followed by Holm‐Šídák correction, was performed to compare cytokine responses to medium (MOI 0) and highest virion concentration (MOI 8; **P < 0.01).

In summary, M11‐CAR‐mediated recognition of virion‐associated gB shapes the target‐specific T‐cell effector functions of M11‐CAR T cells.

## Discussion

In 2010, our group laid the foundation for HCMV‐specific CAR T‐cell research.^19^ We engineered a CAR construct specific for HCMV gB based on the human mAb 27–287 and subsequently demonstrated *in vitro* functionality. Building on the high‐affinity human mAb SM5‐1 (anti‐AD4),^31^, ^32^ Olbrich et al. confirmed *in vivo* efficacy of HCMV gB‐targeting CAR T cells in a humanized mouse model.^20^ While humanized mouse models are valuable tools to overcome the species specificity of HCMV, they remain human‐mouse chimeras and therefore come with limitations including incomplete or variable human immune‐cell reconstitution, restricted spread and pathology of human‐specific pathogens and a disrupted cytokine/chemokine network.^33^, ^34^ Thus, immune responses elicited by human‐derived HCMV‐specific CAR T cells may fail to induce downstream effects. To circumvent these limitations, fully murine CAR constructs can be employed. However, fully murine CAR T‐cell studies are rare and so far, limited to tumor‐specific CARs.^35^, ^36^, ^37^, ^38^ Given the importance of cytokines for viral control, we generated a fully murine second‐generation (4‐1BB) M11‐based CAR construct (Figure 2).^21^ Using MCMV, we assessed the *in vitro* functionality of this MCMV gB‐specific murine CAR under physiological virus–host interactions.

A prerequisite for a functional CAR T‐cell therapy is robust target expression and specific antigen recognition. Therefore, we first demonstrated strong *m55* (gB) transcription in MCMV‐infected MEFs by qPCR and showed specific binding of the mAb M11 to MCMV gB by immunoprecipitation (Figure 1). Using M11‐containing hybridoma supernatant, we visualized target availability and observed high percentages of gB‐positive MEFs by intracellular staining throughout the course of infection; yet, M11‐mediated gB surface staining became evident only at later time points (Figure 1). These results highlight abundant production but differential surface availability of the viral gB and suggest that M11‐based CAR T cells encounter fluctuating target antigen levels. The accuracy of these data is supported by the Croma101 staining: negative Croma101‐surface staining renders unspecific trapping of antibodies, for example, mediated by viral‐FC‐γ receptors, unlikely. Similar percentages of Croma101‐ and M11‐positive cells in intracellular staining further confirm the specificity of M11 for MCMV‐infected cells.^39^

After demonstrating gB recognition by M11 in the context of an MCMV infection, we enabled functional assessment of the M11‐CAR construct by establishing a retroviral transduction protocol of primary murine splenocytes (Figure 2). Within 7 days and without the need for T‐cell purification, our protocol yields high numbers of functional murine CAR T cells using puromycin to select for transduced cells. Kusabuka and colleagues have previously described that puromycin selection of activated and transduced murine splenocytes predominantly gives rise to transduced CD8+ T cells, which is reflected in our retroviral transduction protocol.^40^ CD8+ T cells are key effectors in HCMV as well as MCMV infection.^41^, ^42^, ^43^, ^44^, ^45^ In both mice and humans, CMV‐specific CD8+ T cells accumulate over a lifetime and can make up to 30% of CD8+ cells in the peripheral blood, highlighting the role of CD8+ T cells in long‐term CMV control.^46^, ^47^ Multiple preclinical studies have focused on CD8+ CAR T cells in HIV‐1, HCMV or Hepatitis B virus infection and reported *in vitro* efficacy.^48^, ^49^, ^50^ Notably, Kusabuka et al. showed that purification of CD4+ or CD8+ T cells before activation, retroviral transduction and subsequent puromycin selection increased the yield of CD4+ CAR+ murine T cells, yet decreased the yield of CD8+ CAR+ cells.^40^ These results illustrate the customizability of the retroviral transduction protocol. Considering CAR T‐cell therapies for chronic viral infections, this can be beneficial as certain T‐cell subsets, such as HIV‐1‐susceptible CD4+ cells,^51^ can be reduced or excluded from the final cell product by adapting the manufacturing process.

Applying our transduction protocol, we produced M11‐ and FMC63‐based murine CAR T cells. FMC63, a high‐affinity mAb targeting hCD19, is the foundation of approved cancer immunotherapies (e.g. Kymriah®, NOVARTIS), and a fully murine FMC63‐based CAR served as MCMV‐independent control in our study. First, we demonstrated target‐specific and CAR‐mediated induction of T‐cell effector functions in the absence of MCMV using HEK293T reporter cells expressing either gB or hCD19. Significant lysis of target cells and robust production of IFN‐γ and TNF‐α confirmed the general functionality of our M11‐ and FMC63‐CAR constructs (Figures 2 and 3).

Thereafter, we examined the antiviral effects of our CAR T cells in an *in vitro* MCMV infection model (Figures 3 and 4). To simultaneously enable FMC63‐ and M11‐CAR functionality, we infected hCD19‐expressing MEFs with the MCMV157luc reporter virus. This setup allows us to address (I) whether murine CAR T cells can control an MCMV infection *in vitro*, (II) whether MCMV itself interferes with CAR‐mediated antiviral effects, and (III) whether this applies to CARs targeting MCMV‐related as well as ‐unrelated antigens. Encouragingly, both M11‐ and FMC63‐based CAR T cells mediated a robust reduction in MCMV157luc load in all tested conditions (Figure 3). These results demonstrate that murine CAR T cells can control an MCMV infection *in vitro* and confirm that the M11‐CAR mediates gB recognition and subsequent T‐cell activation under physiologic gB expression. In parallel, we assessed CAR‐mediated lysis of MCMV157luc‐infected hCD19‐MEFs by measuring supernatant Fluc activity 6 h after coculture. Fluc levels were clearly elevated in cocultures with M11‐CAR T cells, but not with nt T cells or FMC63‐CAR T cells. While these results indicate M11‐CAR‐mediated lysis of MCMV157luc‐infected cells, they do not exclude cytotoxic activity of FMC63‐CAR T cells, as Fluc activity is transient and does not accumulate in the supernatant. Thus, supernatant Fluc levels provide insight into recent, but not total, lysis of infected MEFs. The kinetics of M11‐CAR or FMC63‐CAR‐mediated lysis of MCMV‐infected cells remain the subject of ongoing investigation. Here, we provide proof of concept for M11‐CAR T‐cell‐mediated control of MCMV infection in fibroblasts, a well‐established laboratory model for CMV infection. As other murine cell types, including endothelial, epithelial, and myeloid cells, are known to support MCMV infection *in vivo*, our findings establish a robust experimental basis for further investigating M11‐CAR T‐cell functionality in physiologically relevant target cells. Such studies will require cell type‐specific *in vitro* systems, as viral replication and gB expression may differ between cellular contexts.

Beyond virus inhibition and cytotoxicity, we examined CAR‐driven cytokine response to MCMV infection (Figure 4). FMC63‐CAR T cells outperformed M11‐CAR T cells in terms of IFN‐γ and TNF‐α production. Characteristics of a specific CAR T‐cell response are among others determined by the CAR design and the target antigen load.^52^, ^53^, ^54^ Mazzanti et al. showed that CAR constructs consisting of a medium‐length hinge domain surpass constructs with short or long hinge domains in terms of cytotoxicity and cytokine production.^55^ However, as both CARs are based on an identical murine 4‐1BB second‐generation backbone, the difference in cytokine production observed in our MCMV coculture assay may primarily reflect differences in the properties of the respective scFv‐antigen interactions. CAR T cell responses are influenced by several factors, including binding affinity and kinetics, as well as by antigen density and availability. In our experimental setting, the greater cytokine response observed in FMC63 CAR T cells is partially attributable to the high and ubiquitous expression of hCD19, which stands in contrast to the naturally fluctuating expression of MCMV gB. Indeed, fluctuating or low antigen density is a well‐known and frequently addressed issue in CAR T‐cell research. For instance, Majzner and colleagues enhanced the *in vitro* and *in vivo* efficacy of a hCD19‐4‐1BB CAR to low hCD19 tumor cells by exchanging the CD8α‐derived hinge and transmembrane domains with domains derived from CD28.^56^ Such studies open up opportunities to further fine‐tune our M11‐CAR construct in order to recognize MCMV‐infected cells even at low gB density.

We causally linked *in vitro* inhibition of MCMV infection to soluble factors produced by M11‐CAR T cells (Figure 4). Using cell‐free supernatant from M11‐CAR T cell cocultures, we could transfer the antiviral effect. Neutralization of IFN‐γ largely restored viral spread, whereas TNF‐α blockade did not alter inhibition. In both MCMV and HCMV infection, strong synergistic antiviral effects of IFN‐γ and TNF‐α or IFN‐α/β have been demonstrated.^57^, ^58^ While we cannot exclude a contribution of TNF‐α, our results strongly suggest IFN‐γ as the key mediator for M11‐CAR‐driven MCMV inhibition. In line with this, early mouse studies showed that administration of recombinant IFN‐γ significantly reduced mortality in MCMV infection, whereas neutralization of endogenous IFN‐γ increased organ viral load.^59^, ^60^, ^61^

Finally, we demonstrated that the M11 scFv enables binding not only to cell‐associated but also to virion‐associated gB, thereby driving robust cytokine responses (Figure 5). This becomes of particular interest considering our observation of reduced FMC63‐based but increased M11‐based CAR T‐cell responses with increasing MCMV MOI. Since hCD19 antigen availability and contact duration remain unaltered throughout our experiment, exhaustion of FMC63‐based CAR T cells as consequence of higher MOIs is unlikely to explain these results. We rather speculate that MCMV‐infected cells mediated inhibition of FMC63‐based CAR T‐cell responses. Furthermore, we hypothesize that this effect is not apparent in the case of M11‐based CAR T cells because of the additional specific activation by cell‐free virions. Here, cell‐free virions trigger M11‐CAR activation without contributing to the inhibition of T‐cell responses. In this way, direct activation of M11‐CAR T cells via cell‐free virions counteracts inhibition of M11‐CAR T‐cell responses mediated by MCMV‐infected cells. However, clarifying these interactions will require further studies, for example, using MCMV‐mutants deleted in genes associated with inhibition of T‐cell functions and modulation of cell death. CAR‐mediated virion recognition has so far only been described very rarely. In this regard, Kruse et al. discussed the possibility that binding to subviral hepatitis B virus particles (SPVs) could saturate hepatitis B surface antigen (HBsAg)‐specific CAR T cells, limiting their interaction with infected hepatocytes.^62^ Massive production of HBsAg‐displaying SVPs is a hallmark of hepatitis B virus infections.^63^ In contrast, we hypothesize that M11‐CAR‐mediated MCMV virion recognition can establish an antiviral, cytokine‐rich environment around virus‐producing cells without high‐density surface antigen, thereby contributing to non‐cytolytic MCMV control. Being based on the MCMV neutralising mAb M11, M11‐CAR T cells mediated inhibition of MCMV infection; however, the impact of virion recognition needs to be demonstrated *in vivo*. Intriguingly, despite no cytotoxic effect against HBV‐infected cells was observed *in vitro*, the aforementioned HBsAg‐specific CAR T cells reduced the number of HBV‐positive hepatocytes *in vivo*, underscoring the potential of non‐cytolytic, CAR‐mediated viral control.^62^

Treatment of clinically significant HCMV disease in immunocompromised patients aims at viral control rather than virus elimination. In line with this, and despite the pivotal role of CAR‐mediated cytotoxicity in cancer immunotherapy, our *in vitro* characterization of the MCMV‐specific M11‐based CAR highlighted the importance of non‐cytolytic CAR T‐cell functions for viral control. Notably, we showed that the effector functions of virus‐specific CAR T cells can be modulated by recognition of both infected cells and viral particles, opening up a new avenue for fine‐tuning CARs. In conclusion, our fully murine model provides a valuable platform to gain mechanistic insights into virus‐specific CAR T cells and enables the future validation and refinement of CMV‐directed CAR T cells in a physiologically relevant *in vivo* system.

## Methods

### Figures, graphs and statistics

Schemes were generated using BioRender.com, and figures were composed in CorelDraw X6. Graphs were generated and analysed using GraphPad Prism 11. Mean ± standard deviation (SD) is depicted. Based on the experimental design and the distribution of data, statistical tests were selected. Paired Student's *t*‐tests were conducted, followed by Holm‐Šídák correction: *P ≤ 0.05, **P < 0.01, ***P < 0.001, ****P < 0.0001. Statistical tests were only performed when *n* ≥ 3.

### Cell culture

Hybridoma cells (kindly provided by Michael Mach/Marco Thomas, Virology Erlangen) were cultivated in Roswell Park Memorial Institute (RPMI) 1640 medium (11875093, Gibco), supplemented with 10% fetal bovine serum (FBS; A5256701, Gibco), 1× GlutaMAX (35050061, Gibco), 50 μg/mL gentamycin (Sigma‐Aldrich) and 10 mM HEPES (15630056, Gibco) for at least 1 week after thawing. For mAb production, hybridoma cells were pelleted and cultured in 5% Gibco FBS‐containing medium at an initial density of 0.5 × 10^6^/mL. mAb‐containing supernatant was collected at a hybridoma cell viability of 20%. Dead cells were removed by centrifugation, and the mAb‐containing supernatant was stored at −80°C. Murine splenocytes were cultured in lymphocyte medium consisting of RPMI 1640, 10% Gibco FBS, 1× GlutaMAX, 50 μg/mL gentamycin, 10 mM HEPES as well as 50 μM β‐mercaptoethanol (A11080100, AppliChem), 1 mM sodium pyruvate (11360070, Gibco) and 50 U/mL recombinant human interleukin‐2 (rhIL‐2; 11147528001, Roche). For selection and expansion, media were supplemented with 7.5 μg/mL puromycin (sc‐108071B, Santa Cruz Biotechnology) and 10 ng/mL rhIL‐7 (200–07, Peprotech) and recombinant mouse IL‐15 (rmIL‐15; 210‐15, Peprotech). Human embryonic kidney 293T (HEK293T, CRL‐3216; ATCC) cells were cultured in Dulbecco's Modified Eagle Medium (DMEM; 11960‐044, Gibco) containing 10% FBS (AC‐SM‐0173, Anprotec), 1× GlutaMAX, gentamycin (50 μg/mL), HEPES (10 mM) and hygromycin B 200 μg/mL (H0654, Sigma‐Aldrich) and/or 2 μg/mL puromycin, depending on the HEK293T cell line. Murine embryonic fibroblasts (MEF (C57BL/6), SCRC‐1008; ATCC) were cultivated in DMEM, 10% Gibco FBS, 1× GlutaMAX, 50 μg/mL gentamycin, 10 mM HEPES as well as 200 μg/mL hygromycin B and/or 2 μg/mL puromycin. Platinum‐E cells (Plat‐E; Cell Biolabs, Inc.) were cultured in DMEM supplemented with 10% Gibco FBS, 1× GlutaMAX, 50 μg/mL gentamycin, 10 mM HEPES as well as 1 μg/mL puromycin and 10 μg/mL blasticidin. Cells were regularly tested negative for Mycoplasma using MycoAlert Plus Mycoplasma Detection Kit (LT07‐318, Lonza Bioscience).

### Virus production, purification and titration

In this study, a bacterial artificial chromosome (BAC)‐based virus derived from the MCMV157luc construct assembled by Messerle et al. was used.^64^ MCMV157luc encodes firefly luciferase (Fluc) instead of the viral natural killer cell‐activating ligand m157.^23^ Our virus is MCK‐2 wild type and was propagated for infection assays as described by Cordsmeier et al.^65^, ^66^ Titres of viral stocks were quantified using an end‐point titration method. Briefly, 2 × 10^4^ MEFs were seeded in a 96‐well plate and infected with serial dilutions of MCMV157luc. Two days later, cells were fixed with 2% para‐formaldehyde (PFA; P6148, Sigma‐Aldrich) and intracellular staining of the immediate early 1 (IE1) protein was performed using Croma101 hybridoma supernatant and AlexaFluor®488‐conjugated anti‐mouse IgG (α‐ms‐AF488, A‐11029, Invitrogen™) in buffer containing 5% Gibco FBS and 0.5% Saponin (47036, Sigma‐Aldrich). The staining was analysed using a Nikon eclipse Ts2 microscope, and viral titres were calculated according to Reed and Muench.^67^

### Generation of the MCMV gB model

The model of the MCMV gB structure was generated by standard homology modelling procedures using the program MODELLER 9.10,^68^ based on a sequence alignment with the template structures of HCMV gB (PDB: 5CXF and 7KDP). Loop regions that are not resolved in the reference structures were excluded from modelling. Structure analysis and visualization was performed with RasMol^69^ and RasTop (https://www.geneinfinity.org/rastop/).

### Quantitative reverse transcription polymerase chain reaction (RT‐qPCR)

MEFs were infected with MCMV157luc (MOI 4) for 24 h. Supernatant was discarded, cells were washed with PBS, and RNA was extracted using TRIzol (15 596 018, Invitrogen™). RT‐qPCR was performed using the Luna® Universal Probe One‐Step RT‐qPCR Kit (E3006, NEB) and measured in an Applied Biosystems 7500 Real‐Time PCR system.

*m123*:

Probe: 5′/5HEX/AGGTGAGGA/ZEN/GCGTTCTGTTGT/3IABkFQ/3′;

Primer1: 5′/GTTGATGGCTGATTGATAGT/3′;

Primer2: 5′/TATGATAACAGCGACATGG/3′.

*m55*:

Probe: 5′/56/FAM/CTTTCTCGG/ZEN/TGCGTTGGCTGT/3IABkFQ/3′;

Primer1: 5′/TCTAGTCGCTTTCTGCTG/3′;

Primer2: 5′/GGACTAGCGATAAAAAGGG/3′.

*Hrpt1*:

Probe: 5′/5HEX/TGGTGAAAA/ZEN/GGACCTCTCGAAGT/3IABkFQ/3′;

Primer1: 5′/GTACTCATTATAGTCAAGGGCATATCC/3′;

Primer2: 5′/AAATGGTTAAGGTTGCAAG/3′.

### Immunoprecipitation

Murine embryonic fibroblasts were infected with MCMV157luc (MOI 4) for 4 days. Cells were scraped off, pelleted and the supernatant was discarded. After lysis (RIPA buffer) and sonification, volumes corresponding to 300 ng protein (Pierce™ BCA Protein Assay Kit, 23225, Thermo Scientific™) were incubated overnight with 200 μL M11‐containing hybridoma supernatant. Thereafter, 25 μL of Dynabeads™ Pan Mouse IgG (11 041, Invitrogen™) were added for 2 h. After washing (PBS) in a DynaMag‐2™ (12321D, Invitrogen™), the antibody‐protein complex was eluted using 30 μL 0.05 mM glycine (pH 2–2.5; in water). SDS‐Page was performed and protein bands were detected by colloidal Coomassie blue staining and the INTAS advanced fluorescence imager.

### Immunofluorescence analyses of MCMV157luc‐infected MEFs

2 × 10^4^ MEFs were seeded in a 96‐well plate, infected with MCMV157luc (MOI 0.5–8) and incubated for up to 4 days in a wet chamber. For immunohistochemical staining, medium was removed and MEFs were fixed (2% PFA). Surface (PBSo, 5% Gibco FBS) and intracellular (PBSo, 5% Gibco FBS, 0.5% Saponin) staining was performed using Croma101 or M11 hybridoma supernatant and α‐ms‐AF488. Nuclei were stained with Hoechst33342 (H1399, Thermo Scientific™). Staining was captured using the ImageExpress Pico (Molecular Devices; 20x magnification; exposure times: Hoechst33342, 12–15 milliseconds (ms); α‐ms‐AF488, 2000 ms; analysis settings: Hoechst33342, intensity 80, width 4–40; α‐ms‐AF488, intensity 500–1000, width 5–50; α‐ms‐AF488 only staining served as background control). Images were processed with ImageJ2.

### Generation of CAR constructs

Single chain variable fragments (scFv) were synthetized (gBlocks®, IDT) in V_L_ > V_H_ orientation, containing a (G_4_S)_3_ linker. A pUC vector containing the signal peptide, hinge and transmembrane domain of murine CD8α (GenBank: NP_001074579.1; amino acids (aa) M1‐A27 and T152‐C218) linked to the intracellular domain of murine 4‐1BB (GenBank: NP_001070977.1; aa K212‐L256) and CD3ζ (GenBank: NP_001106862.1; aa R52‐R164) was used as cloning vector. The vector was opened between CD8α signal peptide and CD8α hinge domain, and the scFv gBlock was inserted via Gibson assembly. For surface detection, a Flag tag was incorporated between scFv and CD8α hinge domain.^70^ Via DNA restriction and ligation, full‐length CARs were cloned into the pMSCV‐IRES‐Puro resulting in the pMSCV‐M11FBBz and pMSCV‐FMC63FBBz plasmids. In addition, a pMSCV‐eGFP‐IRES‐Puro vector was produced.^28^ This vector was used to visualize enrichment of transduced cells by capturing eGFP signal using the ImageXpress Pico (4x magnification; eGFP filter exposure time: d3 + 4 2000 ms; d7 1000 ms).

### Generation of target cell lines

The M11 target protein gB, the FMC63 target protein human CD19 (hCD19) as well as the molecular reporter nLuc were cloned into lentiviral expression vectors. The resulting plasmids pLV‐EIP‐gB, pLV‐EIP‐CD19 and pLV‐EIH‐nLuc were used for transduction of HEK293T and MEF cells, resulting in HEK293TnLuc, HEK293TnLuc‐gB, HEK293TnLuc‐hCD19 and hCD19‐MEF. Stable transduced cells were selected using the respective antibiotics as described by Bednar^28^ and Menschikowski et al.^29^

### Retroviral transduction of primary murine splenocytes

CAR‐encoding ecotropic retroviruses were produced by transfection of Platinum‐E cells (RV‐101, Cell Biolabs) with the pMSCV vector (2 μg) and 1 μg of the pCL‐Eco plasmid (#12371; Addgene) using polyethyleneimine (PEI; 3 mg/1 μg DNA; 23 966, Polysciences). Two and three days after transfection, retrovirus‐containing supernatant was harvested and pooled. A 12‐well plate was coated with 5 μg/mL goat anti‐hamster IgG (H + L) antibody (31115, Invitrogen™) per well overnight at 4°C. On the next day, splenocytes from a C57BL/6 mouse (027, Charles River) were isolated and erythrocyte lysis was performed. 12 × 10^6^ splenocytes were activated in lymphocyte media supplemented with 2 μg/mL hamster anti‐mouse‐CD3ε (145‐2C11; BioLegend) and hamster anti‐mouse‐CD28 (37.51; BioLegend) and added to the coated 12‐well plate. After overnight incubation, the pre‐conditioned medium was carefully removed from the cells, pooled and mixed with fresh lymphocyte medium lacking antibodies. Retrovirus‐containing Plat‐E supernatant and 10 μg/mL polybrene (TR‐1003; Sigma‐Aldrich) were added to the activated lymphocytes and spinfection was performed at 800 × g and 32°C for 1.5 h. Afterwards, cells were incubated for 4 h under cell culture conditions. Subsequently, viral supernatant was exchanged with the pre‐conditioned medium. On the next day, medium was replaced and cells were transferred to a new, uncoated 12‐well plate. Two days post transduction, cells were resuspended in selection and expansion media containing 7.5 μg/mL puromycin and 10 ng/mL rhIL‐7 and rmIL‐15. Medium was exchanged as necessary and cells were used for coculture assays on day 7. To generate non‐transduced (nt) T cells, a proportion of each donor's splenocytes was processed in parallel but was not subjected to puro selection.

### Antibody staining and flow cytometric analysis

On day 4 and 7 of the transduction protocol, murine cells were stained using biotin‐conjugated mouse anti‐Flag antibody (BioM2; F9291, Sigma‐Aldrich), AlexaFluor®488‐conjugated streptavidin (AB_2337249; Jackson ImmunoReserach), SuperBright™600‐conjugated rat anti‐mouse CD4 (RM4‐5; Invitrogen™), allophycocyanin (APC)‐conjugated rat anti‐mouse CD8α (53‐6.7; BioLegend) and Fixable viability dye APC‐eFluor®780 (65‐0865‐14, eBiosciences™) in FACS buffer (PBSo +10% Gibco FBS). Cells were recorded using the Attune NxT, and data were analysed with FlowJo 10.8.1.

### HEK293TnLuc coculture assay

2 × 10^4^ HEK293TnLuc reporter cells were seeded per well of a 96‐well plate. The next day, 1 × 10^5^ (CAR) T cells were added (effector‐to‐target‐ratio 5:1). 24 h after coculture, nLuc activity was assessed in 50 μL of cell culture supernatant by addition of 100 μL nLuc‐buffer^28^ and luminescence was measured with the Berthold Orion‐II Microplate luminometer. As a maximum lysis control, cells were treated with 0.5% saponin. The remaining supernatant was stored at −20°C until detection of IFN‐γ and TNF‐α concentrations (ELISA MAX™ Deluxe Set Mouse IFN‐γ or TNF‐α, Biolegend).

### MCMV157luc coculture assay

2 × 10^4^ hCD19‐MEFs per well were seeded in a 96‐well plate. On the next day, cells were infected with MCMV157luc (MOI 0.5–8) and incubated for up to 5 days in a wet chamber. On the day of coculture, medium was replaced and 5 × 10^4^ (CAR) T cells were added. After 6 h of coculture, Fluc activity was assessed in the cell culture supernatant, using one‐fifth of the total volume and 100 μL Fluc‐buffer.^28^ After 24 h of coculture, the cell supernatant was stored at −20°C until detection of IFN‐γ and TNF‐α levels. The cell pellet was lysed in 50 μL 1× passive lysis buffer (E1941, Promega) for 15 min at room temperature and Fluc activity was assessed in 10 μL lysate using 100 μL Fluc‐buffer.

### Supernatant transfer assay

Cell‐free supernatant from the MCMVluc157 coculture assay was transferred to 2‐day‐infected MEFs (MOI 0.5). Per well, 30 ng recombinant mouse IFN‐γ (315‐05, PeproTech) or 10 ng TNF‐α (315‐01A, PeproTech) were used as controls. Per well, 1.5 μg neutralizing IFN‐γ (XMG1.2; BioLegend) or 0.5 μg neutralizing TNF‐α (MP6‐XT22; eBioscience™) antibody were used. Fluc activity was assessed 48 h after supernatant transfer using 10 μL of the MEF lysate and 100 μL Fluc‐buffer.

### Quantitative polymerase chain reaction (qPCR)

MCMV157luc coculture supernatant was digested with proteinase K (final concentration 0.136 mg/mL; PCR‐grade, 3115828001, Sigma‐Aldrich) for 1 h at 56°C, followed by enzyme inactivation at 95°C. qPCR was performed using Luna® Universal Probe qPCR Master Mix (M3004, NEB). The PCR reaction was performed and measured in an Applied Biosystems 7500 Real‐Time PCR system (Applied Biosystems, Waltham, MA, USA). Pseudo‐fold change was calculated as: pseudo‐fold change = 2^(Ct_MOI0,5–Ct_sample)^.

*m55*:

Probe: 5′/56/FAM/CTGGCATCC/ZEN/AGGAAAGGCTTGGTG/3IABkFQ/3′;

Primer1: 5′/GCATGGGCCAAGACTGATTGTTCA/3′;

Primer2: 5′/ACCACATGTGTGGATACGCTCTCA/3′.

### Virion‐recognition assay

5 × 10^4^ (CAR) T cells were incubated with increasing MOI of MCMV157luc (MOI 0.5–8) purified by sucrose‐gradient ultracentrifugation. Briefly, cell debris was removed from supernatant by centrifugation (1500 × g, 20 min, RT). Subsequently, MCMVm157luc was pelletized by ultracentrifugation (33 000 × g, 3 h, 4°C). The pellet was resuspended in 1 mL medium and purified via sucrose cushion ultracentrifugation the virus pellet was washed in PBSo and pelletized by ultracentrifugation (70 000 × g, 30 min, 4°C). Finally, the MCMVm157luc virus pellet was incubated overnight (4°C) in 300 μL VSB before being resuspended using a syringe and stored at −80°C on the next day. As a positive control, T cells were activated with 1 μg/mL anti‐mouse CD3 and CD28 antibodies. After 24 h, the supernatant was stored at −20°C until detection of IFN‐γ and TNF‐α concentrations.

## Author contributions

**Sabrina Kübel:** Conceptualization; methodology; writing – review and editing; writing – original draft; visualization. **Christopher Garçon:** Methodology; writing – review and editing. **Hanna Menschikowski:** Methodology. **Arne Cordsmeier:** Methodology; writing – review and editing. **Jens‐Christian Albrecht:** Methodology. **Friederike Oltmanns:** Methodology; writing – review and editing. **Larissa Bauer:** Methodology. **Manuel Hermann:** Methodology; writing – review and editing. **Kristina Holdt:** Methodology. **Heinrich Sticht:** Visualization. **Marco Thomas:** Methodology. **Thomas H Winkler:** Methodology. **Brigitte Scholz:** Methodology. **Doris Jungnickl:** Methodology. **Armin Ensser:** Conceptualization; supervision; project administration; writing – review and editing.

## Conflict of interest

The authors declare no conflict of interest.

## Ethical approval

Spleens were obtained from mice humanely euthanized under licence TS 7/2024 Virologie, Friedrich‐Alexander‐Universität Erlangen‐Nürnberg.

## Supporting information


Supplementary figure 1
Supplementary figure 2
Supplementary figure 3
Supplementary figure 4
Supplementary figure 5

## Data Availability

The authors confirm all supporting data, code and protocols have been provided within the article or through Supplementary data files.
