# Supplementary material for: M11‐CAR T cells inhibit murine cytomegalovirus in vitro upon recognition of virion‐ and cell‐associated glycoprotein B
Source: Clin Transl Immunology. 2026 Nov 3;15(11):e70128. doi: 10.1002/cti2.70128 (PMC13730814; doi:10.1002/cti2.70128)
Supplement: Supplementary file 1 [file CTI2-15-e70128-s001.docx]

**Supplementary Figure 1**

**Supplementary Figure 2**

**Supplementary Figure 3**

**Supplementary Figure 4**

**Supplementary Figure 5**
